# Oncolytic herpes simplex virus delivery of dual CAR targets of CD19 and BCMA as well as immunomodulators to enhance therapeutic efficacy in solid tumors combined with CAR T cell therapy

**DOI:** 10.3389/fonc.2022.1037934

**Published:** 2022-10-24

**Authors:** Yuanyuan Liu, Yanxin Zheng, Tianyi Deng, Yue Huang, Ziwen Liu, Borui Zhan, Xusha Zhou, Runbin Yan, Jiangtao Ren, Yun Xing, Guixing Wu, Biao Zheng, Guang Hu, Wen Wang, Yonghong Liu, Jing Zhao, Xiaoqing Chen, Grace Guoying Zhou

**Affiliations:** ^1^ ImmVira Co., Ltd., Shenzhen, China; ^2^ Nanjing Bioheng Biotech Co., Ltd., Nanjing, China; ^3^ IASO Biotherapeutics Co., Ltd., Shanghai, China

**Keywords:** oHSV, CD19, BCMA, CAR T-cell treatment, solid tumor

## Abstract

**Background:**

The CAR T-cell therapy is a promising approach to treating hematologic malignancies. However, the application in solid tumors still has many tough challenges, including heterogenicity in antigen expressions and immunosuppressive tumor microenvironment (TME). As a new cancer treatment modality, oncolytic virotherapy can be engineered to circumvent these obstacles for CAR T cell therapy in solid tumors.

**Methods:**

In this study, an oHSV T7011 is engineered to drive ectopic expression of dual-antigens, extracellular domains of CD19 and BCMA, on the solid tumor cell surface to be targeted by approved CAR T cells. In addition, multiple immunomodulators, CCL5, IL-12, and anti-PD-1 antibody are also included to modulate the TME. The antitumor activities of T7011 in combination with CD19 or BCMA CAR T-cell were evaluated *in vitro* and *in vivo*.

**Results:**

The expression of CD19 or BMCA on the tumor cell surface could be detected after T7011 infection. The level of CCL5 in TME was also increased. Efficacy studies demonstrated that combination with T7011 and CAR-T^CD19^ or CAR-T^BCMA^ cells showed significant synergistic anti-tumor responses in several solid tumor models.

**Conclusion:**

These studies indicated that the new generation of oHSV T7011 can be a promising combinational therapy with CD19 or BCMA-specific CAR T cells for the treatment of a broad range of solid tumors.

## Introduction

Chimeric antigen receptor (CAR) T cell therapy represents a new and innovative approach to cancer immunotherapy. In 2017, FDA approved the first CAR T cell therapy, CD19-targeted, used for the treatment of B cell acute lymphoblastic leukemia and acute lymphoblastic leukemia (ALL) (1). To date, FDA has approved four CD19-targeted and two BCMA-targeted CAR T cell therapies. The indications involved in these CAR T cell therapies covered blood cancers and bone marrow cancers (2, 3). However, the clinical efficacy of CAR T cell therapy remains limited in solid tumors because of several challenges. Firstly, the solid tumor stroma-like extracellular matrix creates a physical barrier for CAR T cells (4). Secondly, the antigens expressed on tumor cells are heterogeneous and may also express on normal cells, causing on-target off-tumor toxicity (5). Last but not least, the inhibitory tumor microenvironment (TME) including immunosuppressive cells, cytokines as well as immune checkpoints suppress the activation and function of infiltrated effector T cells (6). Therefore, to address these obstacles that prevent translating CAR T cell therapy to solid tumors, new approaches to introduce validated targets in approved CAR T cell therapy specifically to tumor cells and some cytokines or chemokines to TME could enable these CAR T cells to hit intractable solid tumors.

Oncolytic virus (OV) is a new promising therapeutic agent for solid tumors. Among them, oncolytic herpes simplex virus (oHSV) itself can direct tumor cell lysis, with no damage to healthy cells, which contributes to breaking the first barrier for CAR T solid tumor therapy. With genetic engineering manipulation on viral genome, oHSV can arm with some payloads, e.g. cytokines, chemokines, and immune checkpoints inhibitors to convert “cold “ into “hot tumors”.

Talimogene laherparepvec (T-VEC), the first clinically approved OV, is a genetically modified oHSV expressing granulocyte-macrophage colony-stimulating factor (GM-CSF), which is used for melanoma treatment (7). oHSV T3011, constructed with the expression of IL-12 and anti-PD-1 antibody (8), is in phase II trial (NCT04370587) to test its efficacy in advanced or metastatic solid tumors *via* intratumoral injection. What’s more, T3011 has been proven to be safe and effective by intravenous injection in the preclinical study and now has entered phase I clinical study (NCT04780217) in both USA and China. Not only for OV, but CAR T cells are also engineered to express and deliver several types of therapeutic factors. Armed Muc16-directed T cells modified to secrete IL-12 have proven effective in overcoming the immunosuppressive nature of the TME (9). Another mesoCAR T cell expressing anti-PD-1 antibody was used for the treatment of mesothelin positive advanced solid tumor in phase II trial (10). Most importantly, OV can also be modified by expressing and delivering specific CAR antigen onto the tumor surface to increase the targeting of corresponding CAR T cells specifically (11, 12). Anthony et al. have reported that an oncolytic vaccinia virus coding for CD19t has been proved to deliver a truncated non-signaling CD19 protein to solid tumors for combination therapy with CAR T products (13).

Here, based on our clinical-stage product T3011 backbone (8), we developed an oHSV T7011, delivering dual-blood cancer antigens truncated-CD19 and BCMA on the solid tumor cell surface to promote activation and tumor killing by CD19-specific CAR T (CAR-T^CD19^) and BCMA-specific CAR T (CAR-T^BCMA^) cells. The engineered oHSV also expresses chemokine CCL5, which will help to attract endogenous tumor infiltration T cells and CAR T cells to traffic to the tumor sites (14). Once in the tumor, CAR T cells need to proliferate efficiently and persist until the entirety of the tumor is eliminated. At this step, the payloads of oHSV T7011, e.g. IL-12 and anti-PD-1 antibody will contribute to overcoming the inhibitory TME, making it more hospitable to CAR T cells, thereby enhancing overall antitumor activity. To our knowledge, this is the first time that oncolytic herpes simplex virus has been engineered to deliver dual-blood tumor antigens on solid tumors to activate the tumor killing by CD19 and BCMA-specific CAR T cells. The *in vitro* and *in vivo* studies showed synergistic anti-tumor responses by combined treatment with oHSV T7011 and CAR-T^CD19^ cells as well as CAR-T^BCMA^ cells.

## Material and methods

### Cells and viruses

Vero cells were purchased from the American Type Culture Collection (ATCC, Cat. CCL-81, ATCC, USA) and cultured in Dulbecco’s Modified Eagle’s Medium (DMEM, Cat. C11995500BT, Life Technologies, USA) supplemented with 5% newborn calf serum (NBCS, Cat. NCD500, ExCell Bio, China). HEp-2, A375 cells were purchased from the Nan Jing Cobioer Bioscience Co., Ltd, China and cultured in DMEM supplemented with 10% fetal bovine serum (FBS, Cat.10270-106, Gibco, USA). PC-3 and ECA109 cells were purchased from BeNa Culture Collection and cultured in RPMI-1640 containing 10% FBS. CD19, BCMA specific CAR T and normal T cells used for *in vitro* studies were purchased from Jiangsu Tuohong Biotechnology Co., Ltd. The above three T cell lines were cultured in X VIVO 15 (Cat. Q4-418Q, LONZA, Swiss) supplemented with 10% FBS and 300U/mL IL-2 (Cat. Z003618-1, GenScript, China). T cell isolation, lentivirus production and transduction, and *ex vivo* expansion of CD19 or BCMA specific CAR T cells used for *in-vivo* studies were performed as previously described (15, 16). All cells were incubated at 37°C in a humidified atmosphere of 5% CO_2_. HSV-1(F) is the prototype HSV-1 strain used in this laboratory (17). Oncolytic herpes simplex virus (oHSV) T3011 was constructed and reported in the previous study (8).

### Generation of recombinant oncolytic herpes simplex virus T7011

The oHSV T7011 was developed with aid of Bacterial Artificial Chromosomes (BAC) technology. The BAC encoding the HSV-1 (F) DNA was reported elsewhere (18). The payload genes within T7011 contain IL-12, anti-PD-1 antibody, CCL5, and two human truncated non-signaling variants of tumor associate antigens (TAAs) CD19 and BCMA. The coding sequences of CD19, BCMA and CCL5 were linked by T2A self-cleaving peptide sequence (GGAAGCGGAGAGGGCAGAGGAAGTCTGCTAACATGCGGTGACGTCGAGGAGAATCCTGGACCT) to generate a tri-cistronic cassette, which encodes the multiple proteins within one open reading frame driven by HSV-1 immediate early gene promoter (IE4/5 promoter). The T2A-linked expression cassette is inserted between *U_L_37* and *U_L_38* genes. The DNA fragment comprising CD19, BCMA, CCL5, and flanking sequences was ligated into pKO5 at the sites of XbaI and PacI to generate the pKO7011 plasmid. pKO7011 plasmid was then transfected into Escherichia coli harboring BAC-T3011 by electroporation to generate BAC-T7011. Construction of BAC-T3011 was done as reported elsewhere (8). T7011 virus was obtained by transfection with corresponding BAC-T7011 plasmids followed by several steps of plaque purification and amplification in Vero cells.

### Antibodies

The antibodies used in this study included mouse monoclonal anti-CD19 (Cat. AF0093, Beyotime, China), mouse monoclonal biotinylated anti-CD19 (Cat. 302204, Biolegend, USA), rabbit monoclonal recombinant anti-BCMA (Cat. ab253242, Abcam, USA), donkey anti-mouse IgG (H+L) highly cross-adsorbed secondary antibody (Cat. A32766, Invitrogen, USA), goat anti-rabbit IgG (H+L) highly cross-adsorbed secondary antibody (Cat. A-11036, Invitrogen, USA) and Alexa Fluor^®^ 700 mouse anti-Human CD3 (Cat. 557943, BD Pharmingen™, USA).

### Virus titration

Vero cells seeded on T25 flasks were infected with serial 10-fold-diluted virus suspensions in a duplicate manner and incubated at 37°C, 5% CO_2_ for 2 hours. Subsequently, the inoculum was replaced with DMEM medium supplemented with 1% NBCS plus 0.05% (wt/vol) IgG (Cat. S20043008, Weiguang Biological, China) for 72 hours. The cells were fixed with absolute methanol for 5 minutes, rinsed with distilled water, and stained with crystal violet. The plaques were counted, and the virus titer was analyzed.

### ELISA assay

The concentration of anti-human PD-1 antibody was measured as follows: Recombinant his-tagged PD-1 protein (Cat. 10377-H08H, Sino Biological, China) was coated onto the 96-well ELISA plates at 100 ng/well overnight at 4°C. The plates were washed and subsequently blocked with blocking solution (2% bovine serum albumin (BSA), 98% PBST) for 1 hour at room temperature (RT). The plates were then rinsed and exposed to purified anti-PD-1 antibody C1E1-Fab at known concentrations as positive control or to tested samples. After incubation at RT for 2 h, plates were washed and HRP-conjugated goat anti-human Fab (Cat. A0293-1 mL, Sigma, GER; 1:10000) was added. After 1 hour incubation, the plates were washed 3 times and 100 μL tetramethyl benzidine substrate (TMB) substrate was added per well. The plates were developed for 30 min and 100 μL stop solution (Cat. EIA-0031, Beijing Dingguo, China) was added per well to quench the reactions. The optical density (OD) was measured at 450 nm on a BioTek microplate reader. The concentration of anti-human PD-1 antibody in the samples was extrapolated from the standard curve.

The concentrations of IL-12 and CCL5 were measured using ELISA kits (Human IL-12 p70 Quantikine ELISA Kit, Cat. S1200; Human CCL5/RANTES Quantikine ELISA Kit, Cat. SRN00B; R&D Systems, USA) according to the manufacturer’s instructions.

### Immunofluorescence microscopy

*In vitro*: HEp-2 cells (4×10^5^/well) were seeded in coverslip and incubated for 24 hours at 37°C to allow cells to adhere. And then the cells were mock infected or exposed to T7011 at a multiplicity of infection (MOI) of 5 for 1 hour. The inoculum was replaced with fresh culture medium and cultured for the designated time. Cells were rinsed with PBS and fixed with 4% paraformaldehyde for 10 min at RT at the indicated times followed by blocking with 5% skim milk. The cells infected by T7011 were co-stained with an antibody against CD19 (Cat. 302204, Biolegend, USA) as well as those to recombinant anti-BCMA (Cat. ab253242, Abcam, USA). The cells were then incubated with donkey anti-mouse IgG (H+L) highly cross-adsorbed secondary antibody (Cat. A32766, Invitrogen, USA) or goat anti-rabbit IgG (H+L) highly cross-adsorbed secondary antibody (Cat.A-11036, Invitrogen, USA) at RT for 1 hour. Cells were then washed with PBS and embedded in mounting medium (Cat. 8961S, Cell Signaling Technology, USA). The images were captured and processed using a Nikon confocal laser-scanning microscope (HD25, magnification, 120×).

*In vivo*: Tumor tissues were freshly collected and embedded with paraffin according to the conventional process to form FFPE blocks. Then the paraffin-embedded tumor sections (4 μm) were deparaffinized followed by heat-mediated antigen retrieval for 30 min in Dewax solution. After antigen retrieval, tumor sections were permeabilized with 100% methanol, blocked for another 30 min with tris-NaCl (TNB) blocking buffer and then incubated with the mouse monoclonal anti-CD19 (Cat. AF0093, Beyotime, China; 1:100) or rabbit monoclonal recombinant anti-BCMA (Cat. ab253242, Abcam, USA; 1:50) in TNB blocking buffer overnight in a humidified chamber at 4°C. After incubation, tumor sections were washed and incubated with corresponding secondary antibodies. Lastly, the sections were counterstained with DAPI. The images were captured and processed using a NanoZoomer-HT 2.0 Image system for 40× magnification.

### Flow cytometry

Blood and tumor cells were harvested using FCM lysing solution (Cat. LYS03, multiSciences, CN) buffer and tumor dissociation kit (Cat. 130095929, miltenyibiotec, Germany) according to the manufacturer’s instructions. CAR T cell number was determined by flow cytometry using an antibody against human CD3. The samples were incubated with Fc blocker (Cat. 422302, Biolegend, USA) followed by incubation with mouse monoclonal anti-human CD3 labeled with Alexa Fluor^®^ 700 (Cat. 557943, BD Pharmingen™, USA). Stained cells were analyzed using a Gallios (Beckman Coulter) flow cytometer and analyzed with Kaluza software (BD Bioscience) according to the manufacturers’ instructions. The percentage of CAR T cells in blood or tumor tissues was calculated by normalization to total viable cells determined by using the Zombie NIR™ Fixable Viability Kit (Cat. 423106, Biolegend).

### Cell viability assay

CAR T cells or tumor cells were seeded into 96-well plates at a density of 4×10^4^ cells per well or 1×10^4^ cells per well, respectively. After 24-hour of incubation, the cells were infected with or without T7011 at the indicated MOI for designated hours. For the combination studies, the normal control T cells or CAR T cells were added and co-incubated for another 24 hours after viral infection. The viability of the cells was determined by CellTiter-Glo^®^ Luminescent Cell Viability Assay Kit (Cat. G7573, Promega, USA) according to the manufacturer’s instructions.

### Animal experiments

All animal experiments were performed under protocols approved by the Institutional Animal Care and Use Committee of SAFE (Shen Zhen) New Drug Research Technology Company. For human tumor xenograft studies, HEp-2, PC-3 or ECA109 were prepared in DPBS and injected subcutaneously into the flank of NCG mice. Once the mean tumor volumes reached about 80-100 mm^3^, T7011 viruses prepared and diluted in DPBS with 10% glycerin were intratumorally administered at 10^4^ or 10^5^ PFU per mouse for single-dose or multi-dose (on Day 1 and/or Day 7). For combination therapy studies, CAR T cells (5×10^6^ cells/mouse) were prepared in PBS (pH 7.4) and injected intravenously 3 or 7 days after the first dose of T7011 treatment. Tumor growth was monitored three times per week by caliper measurement.

### Statistical analysis

All quantitative data in the study were expressed as mean ± SEM (standard error of means), unless otherwise stated. Statistical differences were analyzed by a two-tailed t-test. P < 0.05 was considered significant unless otherwise stated.

## Results

### Construction and identification of oHSV T7011 delivering truncated CD19 and BCMA to solid tumor *in vitro*

**Figure 1:**
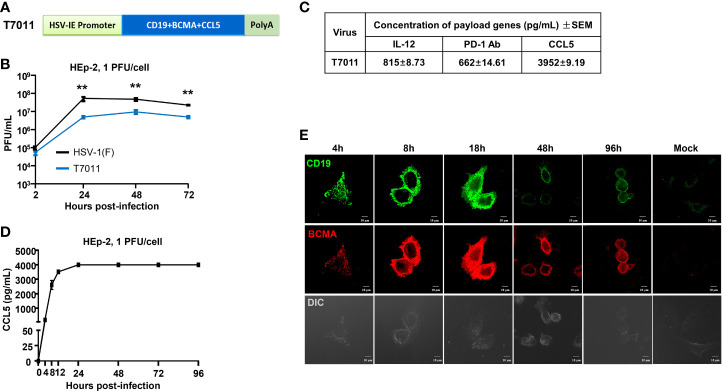
Oncolytic virus T7011 effectively expresses payload genes and delivers truncated CD19 and BCMA to solid tumor surface *in vitro*. **(A)** Schematic representation of the expression cassette of oHSV T7011. Under the control of HSV-1 immediate early (IE) promoter, two truncated non-signaling variant tumor associate antigens CD19 and BCMA, and chemokine CCL5 were inserted between U_L_37 and U_L_38 genes in the T7011 genome, tailed with SV40 polyA. **(B)** Total viral yields were assayed at the indicated time points post infection with T7011. HEp-2 cells were seeded into a 12-well plate at a density of 5×10^5^ cells per well. After overnight incubation, the cells were mock infected or infected with T7011 or HSV-1 (F) at an MOI of 1. After 2-hour incubation, the inoculum was replaced with fresh culture medium. The cell pellets were harvested at 2, 24, 48, and 72-hour post infection (h p.i.) and lysed with three freeze-thaw cycles for titration to detect the virus titer. Statistically significant differences between HSV-1 (F) and T7011 infection groups are indicated by asterisks (**P < 0.01). **(C)** Accumulation of human IL-12, anti-PD-1 antibody, and CCL5 in T7011 infected Vero cells. Vero cells were seeded into a T150 flask at a density of 6×10^6^ cells per flask. After overnight incubation, the cells were infected with 0.01 PFU of T7011 per cell. The supernatant was collected at 48 h p.i. for ELISA assay. Data are shown as means ± standard error of mean (SEM). **(D)** The time-course of CCL5 protein expression in T7011 infected HEp-2 cell. HEp-2 cells were seeded into the T25 flask at a density of 1×10^6^ cells per flask. After overnight incubation, the cells were mock infected or infected at 1 PFU of T7011 per cell. The cell supernatant was harvested at 0, 4, 8, 12, 24, 48, 72, and 96 h p.i. for ELISA assay. Data are shown as means ± SEM. **(E)** The time-course of truncated CD19 and BCMA cell surface localization in T7011 infected HEp-2 cell. HEp-2 cells cultured in coverslip were mock infected or infected with T7011 at 5 PFU per cell. The cells were fixed at indicated hour (h) post infection and then stained with antibodies against CD19 (green) and BCMA (red) as described in *Methods and Materials*. The images including the differential interference contrast (DIC) images were captured and processed using a Nikon confocal laser-scanning microscope. Scale bars = 10 μm.

In this study, recombinant oHSV T7011 was constructed based on the backbone of oHSV T3011 which expresses human IL-12 and anti-PD-1 antibody (8). T7011 was designed to selectively infect a large spectrum of solid tumors and drive tumor-specific expression of CAR-targetable tumor antigens by inserting truncated CD19 and BCMA as well as chemokine CCL5 gene into the loci between *U_L_37* and *U_L_38* with the aid of Bacterial Artificial Chromosome (BAC) technology (**Supplementary Figure 1**). Tandem expression of the above three genes was performed in one open reading frame (ORF) with self-shearing polypeptide T2A under the control of the HSV immediate early (IE) promoter (**Figure 1A**). This construction enables the rapid expression of CD19, BCMA as well as CCL5 after virus entry. As a result, the truncated non-signaling CD19 and BCMA could be delivered to the tumor cell surface as CAR targets and CCL5 would be released into the TEM to infiltrate CAR T cells before oncolytic virus-mediated tumor lysis. As shown in **Figure 1B**, the growth kinetics of T7011 were analyzed in human laryngeal carcinoma (HEp-2) cells and significant lower viral yields were found compared with wild-type HSV (F) infection indicating the genetic attenuation of T7011. The expression of CCL5, as well as IL-12 and anti-PD-1 antibody, was detected *via* ELISA (**Figure 1C**). And the secretion of CCL5 was detected at 4 hours post-infection (h p.i.) at the early time of viral replication cycle and retained for at least 96 hours in the T7011-infected cells (**Figure 1D**). Similarly, the designed targets CD19 and BCMA were efficiently expressed and delivered at HEp-2 cell surface from 4 h p.i. to 96 h p.i. (**Figure 1E**), which indicated that T7011 infection can deliver and display the CAR targets on the tumor cell surface, as well as the release of CCL5 for up to 4 days.

### oHSV T7011 neither replicated effectively nor induced cytotoxicity in CAR T cells

**Figure 2:**
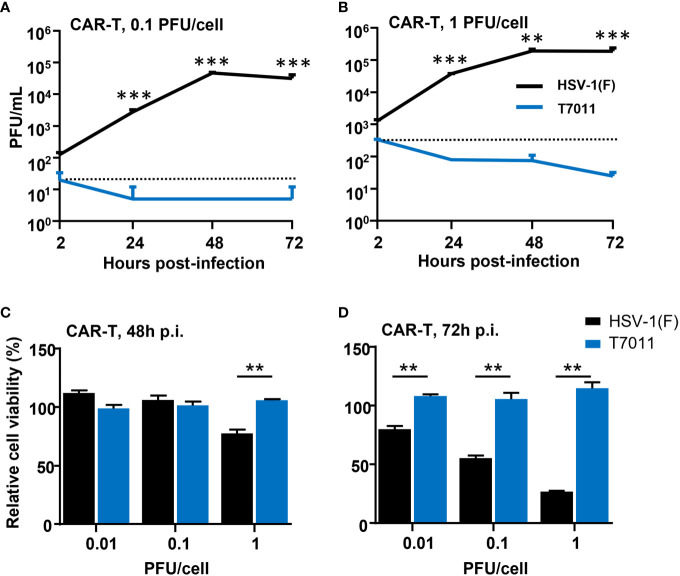
T7011 virus has no infectivity and cell killing activity on CAR T cells. **(A, B)** Viral yields recovered from T7011 or HSV-1 (F) infected CAR-T^CD19^ cells. CAR-T^CD19^ cells seeded in 12-well plates at a density of 5×10^5^ cells per well were infected with or without HSV-1 (F) and T7011 at 0.1 PFU **(A)** or 1 PFU **(B)** per cell. The cell pellets were harvested at 2, 24, 48, and 72 h p.i. and lysed with three freeze-thaw cycles for titration to detect the virus titer. **(C, D)** CAR-T^CD19^ cells seeded in 96-well plates at a density of 4×10^4^ cells per well were mock infected or infected with HSV-1 (F) and T7011 at 0.01, 0.1, or 1 PFU per cell for 48 h **(C)** and 72 h **(D)**. The live cells were determined with CellTiter-Glo^®^ as described in *Methods and Materials*. Results are expressed as the mean of the cell viability ± SEM compared to the mock infection control (as 100%). Statistically significant differences between HSV-1 (F) and T7011 infection groups are indicated by asterisks (**p < 0.01, ***p < 0.001).

As oHSV T7011 infection can continuously deliver and display the CAR targets on the surface of tumor cells, one concern of the combination of T7011 with CAR T cells is whether the CAR T cells would be infected and consequently destroyed. To evaluate whether T7011 infection will induce deleterious effects on CAR T cells, we performed virus infection assay and cytotoxicity assay on CD19 specific CAR T (CAR-T^CD19^) cells. As shown in **Figures 2A, B**, CAR-T^CD19^ cells were infected with wild-type HSV-1 (F) or T7011 at an MOI of 0.1 or 1 and the viral titer showed that the attenuated T7011 is disabled to replicate on CAR T cells, while the wild-type HSV-1(F) virus showed significant higher viral yields compared with T7011. Moreover, there was no cytotoxicity was observed in CAR-T^CD19^ cells which infected with T7011 at varying doses (0.01, 0.1, 1 PFU/cell), while HSV-1 (F) infection could induce significant cytotoxicity in a dose-dependent manner at 72 h p.i. (**Figures 2C, D**). All these results indicated that the attenuated oHSV T7011 showed no infectious activity in the CAR T cells, which will not dampen CAR T function when combined with oHSV T7011.

### Combination with oHSV T7011 and CAR-T^CD19^ or CAR-T^BCMA^ cells enhanced solid tumor cytolysis *in vitro*

**Figure 3:**
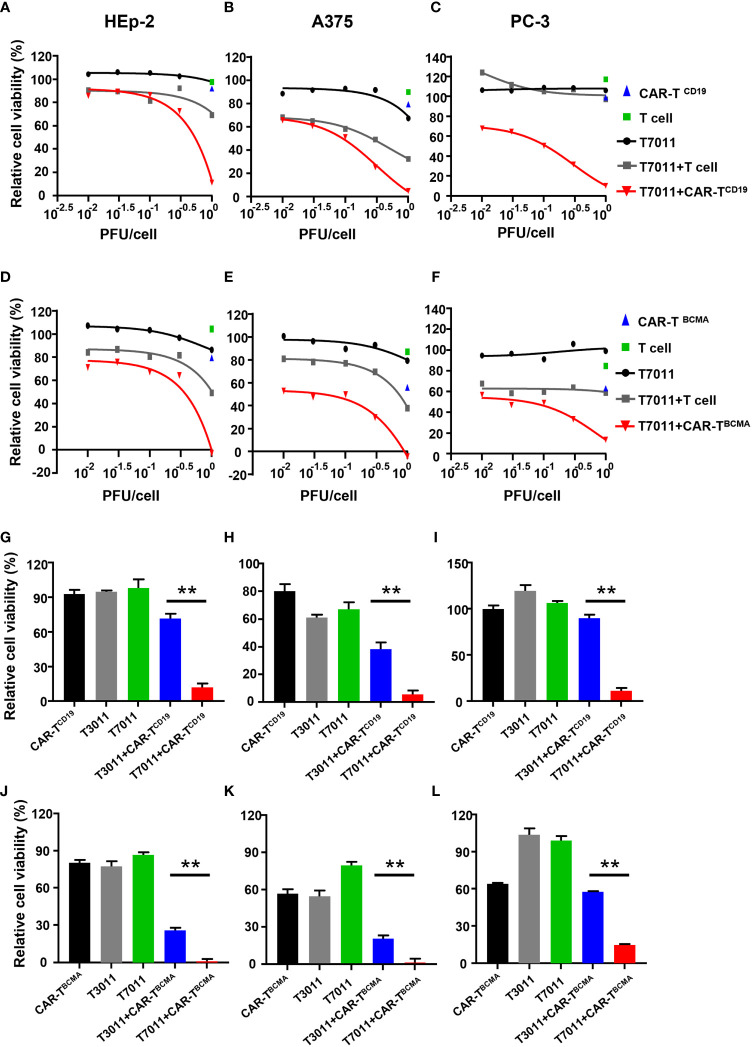
T7011 introduces CD19 and BCMA on tumor cells, which direct activation and cytotoxicity of CAR-T^CD19^ and CAR-T^BCMA^ cells. **(A-F)** HEp-2, A375, and PC-3 cells were infected with T7011 at an indicated MOI of 0.01, 0.03, 0.1, 0.3, or 1 for 24 hours (black line) and then cocultured with untransduced T cells, CD19 specific CAR T cells [CAR-T^CD19^, **(A–C)**], or BCMA specific CAR T cells [CAR-T^BCMA^, **(D-F)**] at an effector: target (E: T) ratio of 4:1 for 24 hours. Cell viability values for tumor cells cocultured with untransduced T cells (green square) or CAR-T^CD19^ cells (blue triangle) are indicated by a single data point. The live cells were determined with CellTiter-Glo^®^. Results are expressed as the mean of the cell viability ± SEM compared to the mock treatment control (as 100%). **(G–L)** HEp-2, A375, and PC-3 cells were cocultured with CD19-specific CAR T cells [CAR-T^CD19^, **(G–I)**], or BCMA-specific CAR T cells [CAR-T^BCMA^, **(J–L)**] at an effector: target (E: T) ratio of 4:1 in the presence or absence of infection of T7011 or T3011 at 1 PFU per cell. The live cells were determined with CellTiter-Glo^®^. Results are expressed as the mean of the cell viability ± SEM compared to the mock treatment control (as 100%). Statistically significant differences are indicated by an asterisk (**p < 0.01).

To assess the activity of CAR-T^CD19^ cells or CAR-T^BCMA^ cells against solid tumor cells when infected with oHSV T7011, human laryngeal carcinoma HEp-2 cells, human melanoma A375 cells, and human prostate carcinoma PC-3 cells were infected with T7011 at varying MOIs for 24 hours and then cocultured with untransduced T cells, CAR-T^CD19^ or CAR-T^BCMA^ cells at an effector: target (E:T) ratio of 4:1 for another 24 hours. As shown in **Figures 3A–C**, compared to the monotreatment groups, the T7011 plus T cell group showed almost no or slightly cell killing ability in PC-3 and HEp-2 cells, and a mild tumor cell killing in A375 cells. While CAR-T^CD19^ treatment showed remarkable cell killing activity against the three tumor cells in an MOI-dependent manner when combined with T7011. For the combination of CAR-T^BCMA^ cells with T7011, a similar pattern was observed as CAR-T^CD19^ cells combined with T7011 (**Figures 3D–F**). To further confirm whether the cell killing was induced specifically by T7011 combined with corresponding CAR T cells, we performed tumor-killing assays on HEp-2, A375, and PC-3 cells with the backbone virus T3011. As expected, **Figures 3G–I** showed that in comparison to CAR-T^CD19^ cells combined with T3011 virus, the CAR-T^CD19^ plus T7011 induced greater killing of tumor cells. A similar result was produced for CAR-T^BCMA^ cells (**Figures 3J–L**). All these results indicated that oHSV T7011 can deliver both CD19- and BCMA-CAR targets to the tumor cells and induce antigen-specific CAR T cell-mediated cytotoxicity.

### oHSV T7011 effectively delivers tumor antigens, CD19 and BCMA as well as CCL5 to the solid tumor *in vivo*

**Figure 4:**
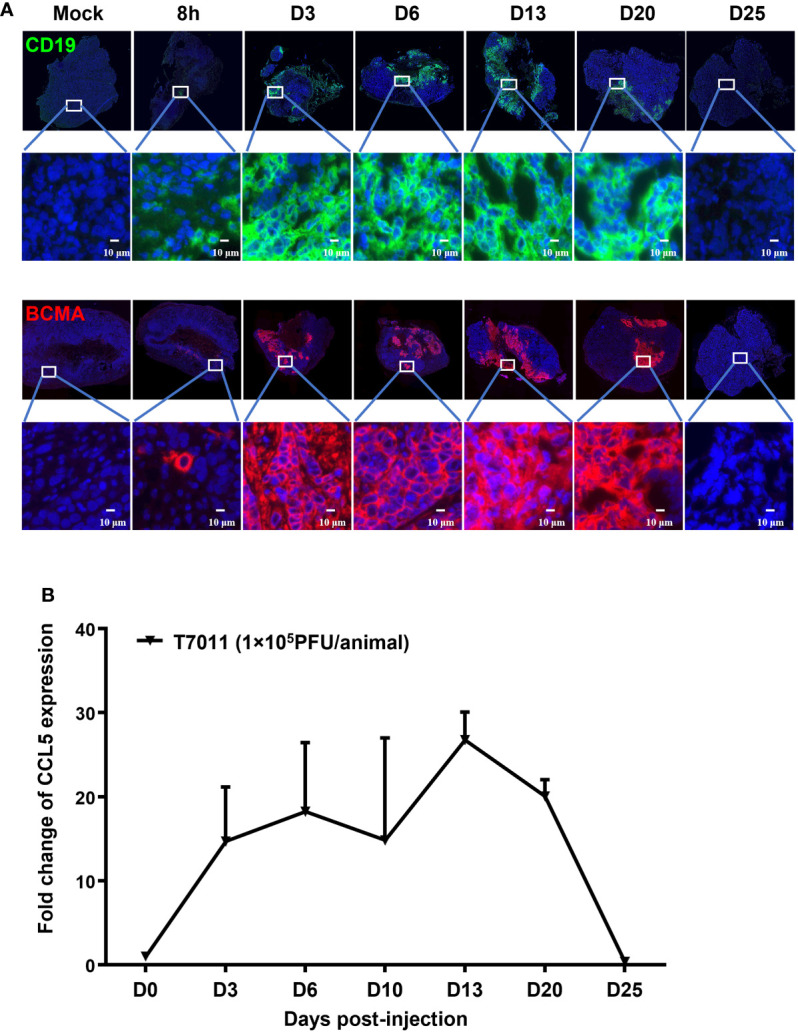
T7011 virus effectively expresses CCL5 in tumor tissue and delivers CD19 and BCMA to solid tumors *in vivo*. **(A)** Visualization of CD19 and BCMA expression *via* immunofluorescence microscopy. HEp-2 xenograft tumor tissues were harvested at pre-dose (Mock), 8h, Day 3 (D3), Day 6 (D6), Day 13 (D13), Day 20 (D20), or Day 25 (D25) after intratumoral injection of T7011 at 1×10^5^ PFU/animal and processed for immunofluorescence assay as described in *Methods and Materials*. The expression of CD19 (green) and BCMA (red) was detected using a designed specific antibody, respectively. The nucleus was stained by DAPI (blue). The images were captured and processed using a NanoZoomer-HT 2.0 Image system. Scale bars = 10 μm. **(B)** Detection of CCL5 expression in tumor tissue by ELISA. HEp-2 xenograft tumor tissues were harvested and homogenized at pre-dose (D0) and Day 3 (D3), Day 6 (D6), Day 13 (D13), Day 20 (D20), or Day 25 (D25) after intratumoral injection of T7011 at 1×10^5^ PFU/animal (n=3 per time point). The CCL5 expression was detected by ELISA assay. The result is reported as fold change after normalization to the D0 group and presented as mean ± SEM.

We have shown that oHSV T7011 can efficiently deliver CD19 and BCMA to solid tumor cells to make them targetable by CD19 or BCMA-specific CAR T cells *in vitro*. Here we assessed whether this same principle holds true *in vivo* model. Immune-deficient mice were implanted with HEp-2 cells and treated *via* intratumoral injection with 1×10^5^ PFU/mouse T7011 at a single dose. As shown in **Figure 4A**, the expression of CD19 and BCMA at the tumor cell surface was detected as early as 8 h p.i. and lasted to 20 days post-infection (d p.i.) at the surface of tumor cells, while failed to detect on 25 d p.i. *in vivo*. It indicated that T7011 virus can efficiently replicate in tumor tissue and continuously deliver CAR targets to tumor cell surface for up to 20 days and thus providing a more optimal therapeutic window for CAR T therapy. Similarly, the CCL5 expression within T7011-infected HEp-2 tumors was detected at a high level from day 3 to day 20 (**Figure 4B**), which suggested the efficient replication of oHSV T7011 occurs, and the payload genes can sustain expression and accumulation.

### Combination therapy of oHSV T7011 and CAR-T^CD19^ cells or CAR-T^BCMA^ cells enhanced anti-tumor activity in several solid tumor xenograft models

**Figure 5:**
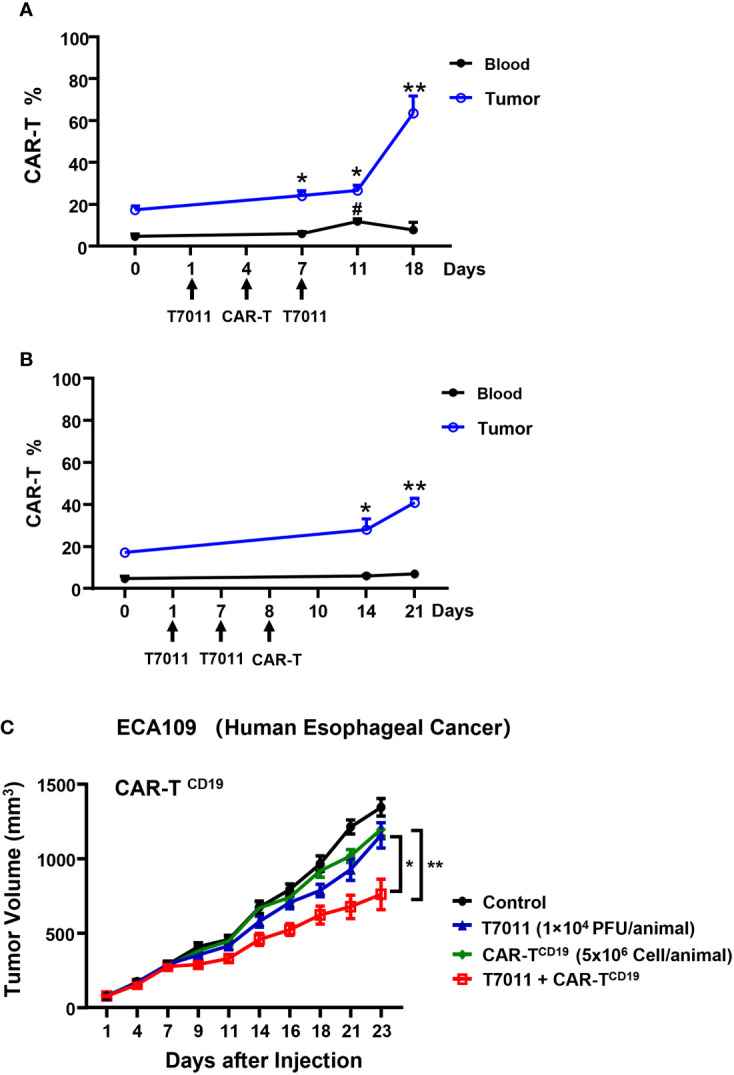
Tumor antigen delivered by T7011 directly activates CAR T cells and sensitizes antitumor activity of CD19-specific CAR T cells against solid tumor cells in human xenograft tumor model. **(A, B)** Percentage of CAR T cells in the blood and tumor tissues in combination with T7011 with different administration sequences. Mice were engrafted with subcutaneous (s.c.) ECA109 tumor cells (3×10^6^ cells), 12 days after inoculation, mice (n=3 per group) were intratumorally (i.t.) injected with T7011 (10^4^ PFU per mouse) on D1, D7, and intravenously (i.v.) injected with CD19-specific CAR T cells (CAR-T^CD19^, 5 × 10^6^ cells) on D8 **(A)** or D4 **(B)**. Tumor tissues and blood were harvested at D0 before dosing or indicated time-points after the first treatment with T7011. The CAR T cells were quantified by flow cytometry as described in *Methods and Materials*. The data are expressed as the mean percentage of human CD3 positive T cells in total viable cells. Statistically significant differences are indicated by asterisks in tumor and by pound signs in blood. (*p < 0.05; **p < 0.01, #p < 0.05). **(C)** Antitumor efficacy of combination therapy of T7011 and CD19-specific CAR T cells in subcutaneous ECA109 tumors model. Mice were engrafted with ECA109 tumors (3×10^6^ cells) subcutaneously (s.c.), 12 days after inoculation, the tumor volume reached 80 mm^3^ were intratumorally (i.t.) injected with T7011 (10^4^ PFU per mouse) on D1 and D7 and intravenously (i.v.) injected with CD19-specific CAR T cells (CAR-T^CD19^, 5 × 10^6^ cells) on D4. Tumor volumes are shown as means ± SEM (n =8 per group). Statistically significant differences are indicated by asterisks (*p < 0.05, **p < 0.01).

To evaluate the therapeutic benefits of oHSV T7011 combined with CAR-T^CD19^ cells or CAR-T^BCMA^ cells *in vivo*, oHSV resistant human esophageal carcinoma ECA109 tumor (19) xenograft model was used. Firstly, we sought to determine the population of CAR T cells in blood and tumor tissue by two different administration sequences. Mice were engrafted subcutaneously with ECA109 tumor cells. After 12 days inoculation, mice were intratumorally (i.t.) injected with T7011 (10^4^ PFU per mouse) on D1, D7, and intravenously (i.v.) injected with CD19-specific CAR T cells (CAR-T^CD19^, 5 × 10^6^ cells) on D4 or D8 (**Figures 5A, B**). The population of CAR T cells was determined at indicated times by flow cytometry. The results showed a significantly higher percentage of CAR-T^CD19^ in tumors detected on 6 and 14-days post-CAR T infusion in both administration sequences when compared with D0 samples respectively (**Figures 5A, B**). And on the D4 CAR T infusion group, the percentage of CAR T in blood samples was detected significantly higher 7-days post-CAR T infusion but did not observe in the D8 infusion group. What’s more, the percentage of CAR-T^CD19^ cells in tumor tissue administrated on D4 was 20% higher than that of D8 (**Figures 5A, B**). All these results indicated that T7011 injection on D1 and D7, and CAR T injection on D4 can efficiently infiltrate CAR T cells into TME and are more likely to induce the optimal CAR T cell-mediated antitumor activity. Consistently, following this administration sequence, the combination therapy of oHSV T7011 and CD19-CAR T cells achieved significant inhibition of tumor growth compared to the monotherapy groups (**Figure 5C**).

**Figure 6:**
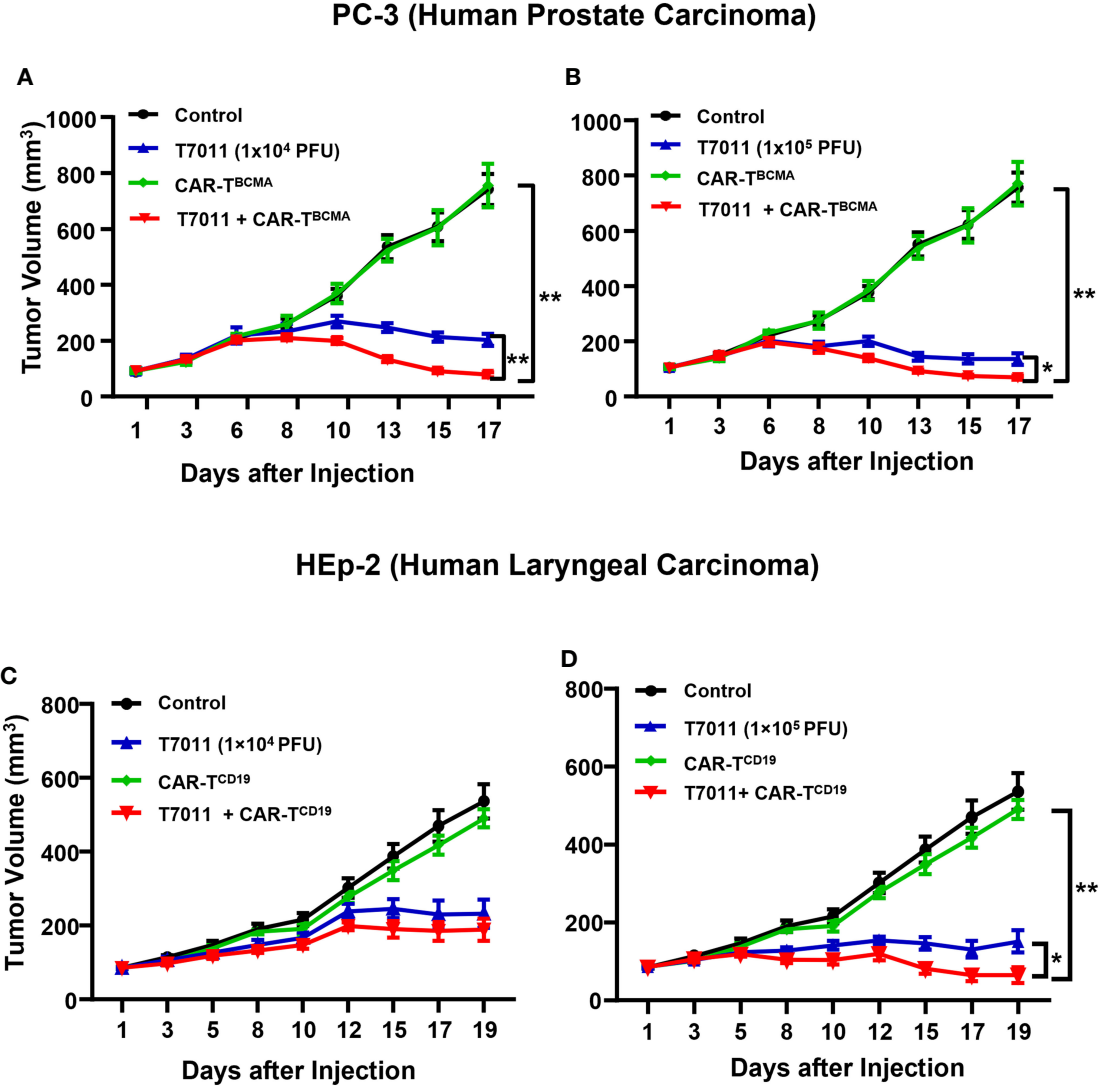
Dose-dependent antitumor activity of combination therapy of T7011 and CAR T cells in human HEp-2 and PC-3 xenograft tumor models. **(A, B)** Mice implanted with PC-3 tumors averaging to 100 mm^3^ were intratumorally injected twice with PBS or T7011 at 10^4^ **(A)** or 10^5^ PFU **(B)** per mouse on D1 and D7. At D4, the mice were intravenously mock injected or injected with 5 × 10^6^ BCMA-specific CAR T cells (CAR-T^BCMA^). Tumor volumes were measured, and the data are shown as means ± SEM (n =8 per group). **(C, D)** Mice implanted with HEp-2 tumors averaging 85 mm^3^ were intratumorally injected twice with PBS or T7011 at 10^4^ **(C)** or 10^5^ PFU **(D)** per mouse on D1 and D7. At D4, the mice were intravenously mock injected or injected with 5 × 10^6^ of CD19-specific CAR T cells (CAR-T^CD19^). Tumor volumes were measured, and the data are shown as means ± SEM (n =8 per group). Statistically significant differences are indicated by an asterisk (*p < 0.05, **p < 0.01).

We also assessed the antitumor efficacy of combination therapy in other PC-3 and HEp-2 tumor models using the above administration sequence. We found that in comparison to the monotreatment, a significant reduction in tumor burden in mice treated with intratumoral T7011 and intravenous BCMA-CAR T cells (**Figures 6A, B**). Similarly, the combination antitumor effect of T7011 and CD19-CAR T cells was achieved in the HEp-2 model in a T7011 dose-dependent manner (**Figures 6C, D**). Altogether, our data showed that the broad applicability of our combinational therapy approach with T7011-mediated delivery of CAR targets, CD19 and BCMA, on solid tumor surface that makes solid tumor targetable by both CD19 and BCMA-specific CAR T cells and exerts enhanced antitumor activity.

## Discussion

To overcome the challenges of CAR T cell therapy used in solid tumor, oHSV expressing CAR targets, cytokine and chemokine as well as immune-checkpoint inhibitor was designed for combined with CAR T cell in this study. In detail, engineered oHSV T7011 was used to deliver dual-blood cancer antigens, the extracellular domain of CD19 and BCMA, to solid tumor surface to activate the tumor-killing by CD19 or BCMA-specific CAR T cells. Meanwhile, the oHSV expressing cytokine IL-12, and chemokine CCL5 as well as checkpoint inhibitor anti-PD-1 antibody, further augment CAR T cell trafficking to the tumor site and their antitumor activities. Definitely, the oHSV itself not only contributes to destroying the physical barrier for the entrance of CAR T cell, but also converts the immunosuppressive TME to a more accommodating place for their stimulation, expansion, and persistence (20). Here, we confirmed that combination with oHSV T7011 and CAR-T^CD19^ or CAR-T^BCMA^ showed synergistic anti-tumor efficacy in both *in vitro* and *in vivo* studies, including human melanoma, esophageal carcinoma, prostate carcinoma, and laryngeal carcinoma models.

Single therapy with CAR-T^CD19^ cell or CAR-T^BCMA^ cell has been approved by FDA in recent years, which is used for the treatment of children and adults with hematological malignancy (1). Engineered oncolytic vaccinia virus or adenoviruses coding for CD19 have been used as an oncolytic tagging system, which can diminish established tumors *in vivo* and prolong mice survival significantly (13, 21, 22). Zhang et al. reported that the pre-clinical data demonstrated that CD19 and BCMA bispecific CAR T cells are effective in eliminating multiple myeloma tumor cells both *in vitro* and *in vivo*. And the first-in-human clinical trial further confirmed the safety and efficacy of CD19-BCMA bispecific CAR T in treating relapsed and refractory multiple myeloma (23). In our study, oHSV T7011 was engineered to deliver both CD19 and BCMA CAR targets to solid tumors. And the construction was to use immediate early promoter of HSV to drive transgene expression. Thus, once T7011 infected the solid tumor cells, the CAR targets will be expressed. We also have proved that three days prior to the CAR T cell infusion, T7011 has already delivered both CD19 and BCMA CAR targets onto tumor cell surface *in vivo*. And on day 4, when CAR T cell was infused, the expression of CAR targets reached the maximum and lasted constantly for 20 days, which allowed CAR T cell to have plenty of time to be activated and impose a lytic effect on tumor cells. In addition, we must mention the on-target, off-tumor toxicities when CAR T cells therapy was used. The CAR targets were delivered by oHSV T7011 which has the same attenuation strategy as T3011 (NCT04370587, NCT04780217) with genetic deletion of 15-kb internal repeat (IR) sequence, which has been well characterized that can restrict virus replication only in normal cells, but not in tumor cells. In other words, CAR targets only accumulated in the infected tumor cells, displaying as the target for CAR T cells.

The CD19 and BCMA delivered to the tumors by oHSV are crucial for CAR T cell therapy. Before CAR T cell arrived at the targeting tumor cells, infused CAR T cells need to pass from the bloodstream to solid tumor regions, penetrate into the tumor stroma and further increase the availability for immune infiltration (24). It has been reported that infection with OVs in tumor cells will change the expression profile of cytokines or chemokines in TME, thus providing more opportunities for optimal CAR T cells penetrance and infiltration in the tumor tissue (20). One Muc16-directed T cell engineered to secrete IL-12, which is in phase I clinical trial (NCT02498912), has shown the enhanced cytotoxic capability of CD8^+^ T cells as well as reduced antigen escape in CAR T cell treatment by recruiting macrophages to the TME (9). In addition to cytokines, numerous chemokines also can mediate immune cell trafficking, and modulation of chemokine signaling has been explored to enhance T cell localization to tumors (25). Some chemokines, such as CCL5 (RANTES), CXCL10 and CCL2 induced by TNF*α*, also have been reported to locally recruit both CAR T cells and endogenous T cells when combined with adenoviral OV and mesothelin-redirected CAR T cells in pancreatic ductal adenocarcinoma model (26).

After the infused CAR T cells were attracted to the tumor regions, one important step is to overcome T cell inhibitory signals present in the TME. Of note, PD-1 as an immune-checkpoint receptor expressed on activated T cells and, when bound by its ligand PD-L1, can induce T cells to adopt an exhausted, ineffective phenotype. Moreover, PD-1 expression has been demonstrated to be a contributing factor to CAR T cell dysfunction (27). Thus, the payload PD-1 antibody expressed in T7011 will help CAR T cells to overwhelm the inhibition induced by PD-1/PD-L1 signal pathway, thus reducing the CAR T cells exhaustion. Meanwhile, at a lower dose of T7011, there was no cell killing of CAR T cells mentioned. It suggested that the proper administration sequence and dose need to be considered in the combination therapy. In addition to PD-1, another combination therapy with EGFRvIII-directed CAR T cell engineered to express anti-CTLA-4 and PD-1 antibodies, is in the second phase I/II clinical trial (NCT03182816), showing improved persistence and activity of T cells in its cancer killing capacity (28).

The oHSV T7011 used in this study can express immune-checkpoint inhibitor anti-PD-1 antibody, cytokine IL-12 and chemokine CCL5. Among them, the expression of anti-PD-1 antibody and IL-12 was consistent with the expression profile reported elsewhere (8) and the constant expression of CCL5 can last for 3 weeks *in vivo.* In this study, we just evaluated T7011-mediated CAR targets and their anti-tumor activity combined with CAR T cell treatment in immunodeficient mice. The bioactivity of the payload immunomodulators including anti-PD-1 antibody, IL-12, and CCL5 need to be further evaluated in immunocompetent murine models in the future, which will provide more comprehensive antitumor efficacy evaluation for combination therapy with T7011 and CAR T. Nevertheless, this study will give some new insights into the clinical development of combination therapy with OV and CAR T therapy for the treatment of solid tumors.

## Data availability statement

The original contributions presented in the study are included in the article/**Supplementary Material**. Further inquiries can be directed to the corresponding authors.

## Ethics statement

The animal study was reviewed and approved by Institutional Animal Care and Use Committee of SAFE (Shen Zhen) New Drug Research Technology Company.

## Author contributions

Conceptualization, JZ, XC and GZ; Formal analysis, YYL, YZ and TD; Funding acquisition, YHL and GZ; Investigation, YYL, YZ, TD, YH, ZL, BoZ and XC; Methodology, YYL, YZ, TD, ZL, BoZ and XZ; Project administration, XC and GZ; Resources, ZL, RY, JR, YX, GW, BiZ, GH, WW and GZ; Supervision, XC and GZ; Validation, YYL, YZ, TD, YH, XZ and RY; Visualization, YYL, YZ, TD, YH and JZ; Writing – original draft, YYL, YZ, JZ and XC; Writing – review & editing, YYL, YZ, YHL, JZ, XC and GZ. All authors contributed to the article and approved the submitted version.

## Funding

This work was partially funded by grant from Young Scientists Fund of National Natural Science Foundation of China 31900136.

## Conflict of interest

Authors YYL, YZ, TD, YH, ZL, BoZ, XZ, RY, YHL, JZ, XC and GZ were employed by ImmVira Co., Ltd, JR, YX and GW were employed by Nanjing Bioheng Biotech Co., Ltd. and BiZ, GH and WW were employed by IASO Biotherapeutics Co., Ltd.

## Publisher’s note

All claims expressed in this article are solely those of the authors and do not necessarily represent those of their affiliated organizations, or those of the publisher, the editors and the reviewers. Any product that may be evaluated in this article, or claim that may be made by its manufacturer, is not guaranteed or endorsed by the publisher.
