# Supplementary material for: Oncolytic herpes simplex virus delivery of dual CAR targets of CD19 and BCMA as well as immunomodulators to enhance therapeutic efficacy in solid tumors combined with CAR T cell therapy
Source: Front Oncol. 2022 Oct 24;12:1037934. doi: 10.3389/fonc.2022.1037934 (PMC9638445; doi:10.3389/fonc.2022.1037934)
Supplement: Supplementary file 1 [file DataSheet_1.docx]

**Supplementary figure**

**Supplementary Fig.1**


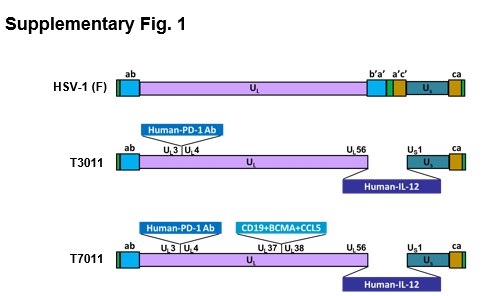


Schematic representation of HSV-1 (F), T3011, and T7011 genomes. HSV-1 (F), a wild-type HSV-1 virus; T3011 is a genetically modified virus that includes deletion of the 15-kb internal repeat (IR) sequence, and insertion of human IL-12. Another exogenous gene anti-human PD-1 antibody is inserted into the genome between *U_L_3* and *U_L_4*; T7011, a genetically modified virus based on the T3011, and has additional genes coding the ectodomain of human blood cancer antigens CD19, BCMA, and full length of CCL5, which introduced into the intergenic region between*U_L_37* and *U_L_38* as one expression cassette.
